# Health Is Still Social: Contemporary Examples in the Age of the Genome

**DOI:** 10.1371/journal.pmed.0030419

**Published:** 2006-10-24

**Authors:** Timothy H Holtz, Seth Holmes, Scott Stonington, Leon Eisenberg

## Abstract

Holtz and colleagues argue that social medicine, including an understanding of the social roots of disease, is as important now as it has ever been.

In 1999, Leon Eisenberg wrote an essay entitled, “Does social medicine still matter in an era of molecular medicine?” [1]. Anticipating the scientific discussion that would accompany the complete mapping of the human genome, followed by hubristic predictions of an end to disease through the introduction of gene-based therapy, Eisenberg reminded his readers of the inherent social basis of disease causation. “The developments in molecular biology highlight the salience of the social environment and underscore the urgency to rectify inequity and injustice. All medicine is inescapably social,” he wrote.

In this Essay, we revisit those concerns and expand them to discuss the current state of scholarship on the social causes of, experiences of, and responses to disease. We contend that social medicine is as important now as it has ever been. The field of social medicine includes various social and cultural studies of health and medicine [2], and in this article, we will focus on one domain of these studies—the social roots of disease—to illustrate the contemporary importance of social medicine.

## Contemporary Examples

The final sequencing of the human genome was announced in 2001 and greeted with great fanfare. Scientists have since cloned the embryo of a sheep, followed by a dog, cow, horse, mule, and mouse. With automated DNA sequencing, the genetic code may soon be cracked for nearly every major animal phylum. Many scientists are hopeful that these developments will eventually produce laboratory-based gene therapies that will cure many human diseases. What is invariably lost in these celebrations of scientific advances is awareness that human social organization is the primary determinant of how diseases are distributed in society, and that much of human disease results from preventable social factors. This inextricable link between social inequality and ill health is seen in nearly every field of medicine.

### Environmental disasters.

As a first example, consider the recent global environmental disasters, which, natural or unnatural, laid bare the inequalities that cut across society. The years 2004 and 2005 were terrible years for “natural” disasters—the Asian tsunami on December 26, 2004 [3], Hurricane Katrina in September 2005 [4], and the Kashmir earthquake in October 2005 [5]. Although nature triggered these events, there was nothing “natural” about the extent to which certain people were more likely to die. All disasters are shaped by the context and hierarchy of human social organization.

The role of human behavior and social organization in determining who was at risk is an ignored but vital aspect of disasters. In a landmark study, sociologist Eric Kleinenberg, researching the Chicago heat wave of 1995, found that the mortality attributed to the disaster could only be fully understood with a “social autopsy” of the event. Without “de-naturalizing” the event, its outcomes and its relationship with social inequalities and local policies remained obscure [6].

**Figure:**
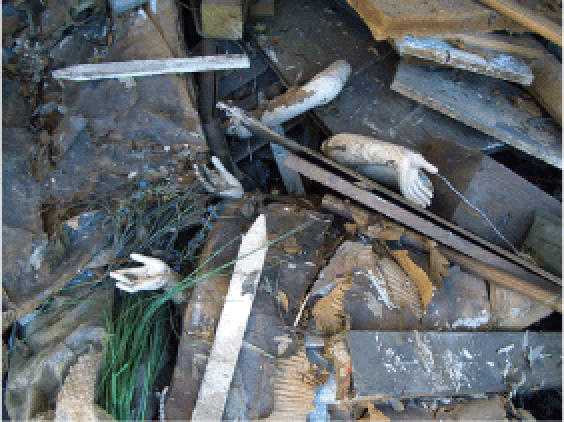
The devastation wrought by Hurricane Katrina exposed underlying social inequalities (Photo: Waving Goodbye, Abigail Hankin)

There is no better illustration of the need for a social autopsy of a disaster than the devastation wrought on urban New Orleans and surrounding communities by Hurricane Katrina [7]. Because of extensive flooding in the city, more than 75% of the city's 500,000 residents became internally displaced virtually overnight. Most of those who could not escape the storm were poor, living in historically economically deprived communities. Many of these communities were also predominately African American, such as the hard-hit Lower Ninth Ward. Most residents who stayed behind and bore the full impact of the category 5 hurricane (it was downgraded to category 3 by the time it hit the coast) did not have the luxury of choosing to leave; they had no option but to stay. They had neither cars nor ready access to transport for evacuation. Who lived or died was more a reflection of available housing and transportation options, not biologic risk factors. The disaster may have been “natural” in a meteorological sense (although some now argue that global warming has increased the probability of hurricanes), but it was the “unnatural” social forces—specifically class and race—that determined who lived and who died.

### Health disparities.

Though Katrina unfolded before our eyes on television, greater tragedy has been playing out for centuries in United States society in morbidity and mortality differentials between socially defined racial groups, particularly between whites and blacks. The overall death rate for African Americans, today, is comparable to the rate for white Americans some 30 years ago. This translates into 100,000 African Americans dying every year (most from chronic diseases) who otherwise would not die if the death rates were similar between the two groups [8]. Disparities in infectious diseases at the beginning of the 20th century are now being replicated in disparities in cardiovascular disease, cancer outcomes, and many other chronic diseases at the beginning of the 21st century [9].

Although the prevalence of heart disease and diabetes is two to three times higher in African Americans than in whites, representative surveys of Caribbean populations of African origin have revealed prevalence rates two to five times lower than those of blacks in America or Britain [10]. This should give pause to those searching for solely biologic explanations of racial disparities in disease. The disparities in these rates in the US are partially due to socially patterned behaviors. The persistent differences are also strongly related to social disparities such as residential segregation, neighborhood quality, and labor conditions that create, contribute to, and exacerbate ill health [9]. In addition, white and black patients presenting with the same signs and symptoms are given significantly different medical care, based on their perceived racial or ethnic identity [11].

Despite pernicious associations between race and inequality, modern medicine pushes ahead with efforts to produce race-specific therapy for diseases such as heart disease and diabetes. In June 2005, the first “race-specific” patented drug (isosorbide dinitrate combined with hydralazine hydrochloride, or BiDil) was approved for the treatment of heart failure in African Americans [12]. The drug is a combination of two generic medications that have been off patent for years and widely used to control heart failure. Through clever marketing, the combination drug was presented to the US Food and Drug Administration as a “race-specific” drug, after initially being denied a patent—the first US drug to be based on a patent formulated in terms of its benefit to a specific racial group. The approval of BiDil presages a trend in the pharmaceutical industry to use race as a proxy “genetic” biologic marker to address health disparities through commercial drug development [13]. What might be called the “desocializing” or “geneticizing” of race has potentially worrisome implications (discussed in [14]).

**Figure:**
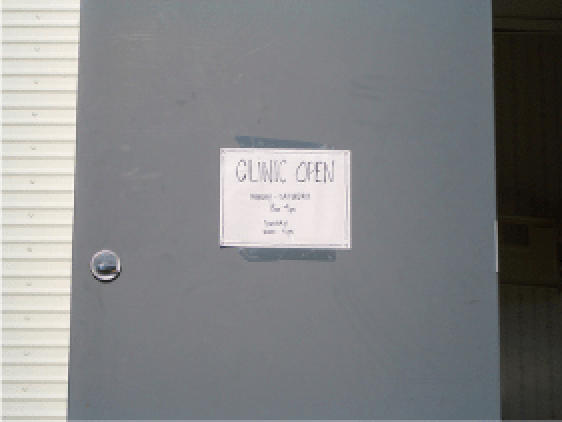
A makeshift clinic serving patients affected by Hurricane Katrina (Photo: Clinic Open, Abigail Hankin)

### Distribution of health care.

Julian Tudor Hart, who served as a primary-care physician for Welsh miners for more than 30 years [15], established a clinical research practice based on medical surveillance of his entire population of 1,900 patients (rather than limiting his purview to the fraction who attended his surgery). From this experience, Hart coined the “Inverse Care Law” [16]: “The availability of good medical care varies inversely with the need for it in the population served. . . [all the] more completely where medical care is most exposed to market forces . . ” Even in a nationalized health system such as in the United Kingdom, resource distribution reflects the social status of communities and determines the health of the people in them [17].

Only 10% of the world's health research funding goes toward the diseases that make up 90% of the global burden, a situation known as the “10/90 gap.” Traditionally, pharmaceutical companies have focused their research on diseases of the rich world. Fortunately there has been a recent surge in the pharmaceutical industry's engagement in developing drugs for the neglected diseases of poverty [18].

## The Foundation for Change in the Field

### Virchow's principles.

In 1848, the German physician Rudolf Virchow laid the foundation for the practice of social medicine, and advocated that medicine be reformed on the basis of three principles: (1) the health of the people is a matter of direct social concern; (2) social and economic conditions have an important effect on health and disease, and these relations must be subjected to scientific investigation; and (3) the measures taken to promote health and to combat disease must be social, as well as medical [19].

In the 150 years since Virchow produced his principles, medicine has strayed from this vision [20]. Despite television images of trapped hurricane survivors searching for food and the knowledge that 44 million Americans (most of them working) do not have health insurance, medical research continues its biomedical trajectory in search of expensive “magic bullets” and more sophisticated interventional technologies, rather than understanding the social determinants of health. The field of medicine needs to return to Virchow's principles and highlight the social, as well as the biological risks, for disease. We need to recognize that relative positions in society affect health, exposure to illness, risk for illness-producing behaviors, and the patient's sense of agency.

Though science has become more interdisciplinary in nature, the disciplines invited to the table do not always include the social sciences. The door is slightly ajar and must be pried open. As Karl Popper reminds us, “We are not students of some subject matter, but students of problems. And problems may cut across the borders of any subject matter or discipline [21].”

Why then is social science often excluded? For one, academic medicine has been preempted by the glamour of technology and by the rewards it brings to those who discover and employ it [22]. For another, social scientists are unwelcome when they discover unpleasant facts, such as life circumstances trumping medical care in determining the health status of populations and that disparities in health care are part of the system rather than oversights [1]. These discoveries not only threaten medical hegemony, but they challenge the larger social order [23].

## The Roots of Disease Are Still Social

We cannot look at the status of a population's health without examining the social context. Consider the risk of exposure, host susceptibility, course of disease, and disease outcome; each is shaped by the social matrix, whether the disease is labeled “infectious,” “genetic,” “metabolic,” “malignant,” or “degenerative.”

The distribution of health and disease in human populations reflects where people live; when in history they live; the air they breathe and the water they drink; what and how much they eat; the energy they expend; the work they do; the status they occupy in the social order and how they are socialized to respond to and identify with or resist this status; who, when, and whether they marry; whether they are socially isolated or rich in friends; the amount and kind of medical care they receive; and whether they are stigmatized when sick or receive care in the community. This is no new discovery. The Hippocratic Treatise “Airs, Waters, Places” enjoins, “whoever wishes to pursue properly the science of medicine” to consider, among other features of the place of practice, “the mode of life . . . of the inhabitants, whether they are heavy drinkers, taking lunch and inactive, or athletic, industrious, eating much and drinking little.”

Contemporary medical indifference to “mode of life” is a legacy of, among other things, the spectacular accomplishments of bacteriology in the late-19th century, which made single causes and single effects the paradigm for medical theory. But the paradigm is flawed. Bacteria do not fully account for disease pathogenesis. Infection by the tubercle bacillus is a necessary, but not sufficient, condition for clinical tuberculosis. Of individuals with a primary infection (evidenced by a positive tuberculin skin test), only a minority ever display the symptoms and signs of clinical disease. Susceptibility to tuberculosis varies not only with age and sex but also with housing and social class [24]. Mortality data for England and Wales show that the death rate from tuberculosis had already fallen by half during the 40 years before Koch discovered the bacillus. During the next 60 years, it fell by more than half again before effective chemotherapy (streptomycin) was introduced [25]. Decreasing morbidity and mortality rates reflected improved living conditions.

We cannot confine our alleviation of suffering to patient biology.

Enormous health disparities exist in this century around the world. Life expectancy ranges from 34 years in Sierra Leone to 82 years in Japan [26]. There is a marked social gradient within countries: households with more wealth, higher incomes, better education, and safer jobs (socioeconomic status) have lower mortality rates. The gap in life expectancy between the most-advantaged and the least-advantaged populations in the US is 20 years. What social conditions give rise to or contribute to increased risk for disease? Unhealthy behaviors and life stressors contribute to and exacerbate disease risk, but “the health of the population is a measure of whether, in the end, that population is benefiting as the result of a set of its social arrangements [26].”

## From Understanding to Action

Michael Marmot, chairman of the World Health Organization's Commission on Social Determinants of Health, said that “if the major determinants of health are social, so must be the remedies. Health status is the best measure of whether a population is thriving” [26].

### Interdiscplinary research.

Complex health problems are insoluble without understanding social context. There is an urgent need for social scientists, and in particular physician social scientists, to participate in interdisciplinary research. Training in existing disciplines should be broadened so that graduates become aware of the concepts and methods at the borders of their fields. Basic scientists should be introduced to clinical problems; clinical investigators should be kept abreast of laboratory disciplines.

### Training physicians in social science.

We need to create the minimum expectation that all physicians be trained as “informed consumers” of social science, able to recognize implications applicable to their own work [27]. Beyond this, our contemporary world needs practitioners dually trained in medicine and the social sciences. We need scholars who can produce social analysis grounded in suffering, and who are committed to ameliorating that suffering. And we need humanist scholars who can unveil the experience of suffering and health care. These physician scholars will be those best suited to guide medical education in the areas of social disparities in health, cultural competency, and beyond.

### Developing a “community-side manner.”

If we want to fulfill our role as medical professionals, we cannot confine our alleviation of suffering to patient biology. Our bedside manner should be extended to an informed “community-side manner” that considers all the social contributing factors to human health.

Currently, the time constraints and scope of medical practice make it difficult for practitioners to develop this skill—to question their own narrow training, to explore and understand the social forces that affect their patients, and to intervene beyond bodies and into social worlds. The version of the Hippocratic Oath most commonly used in medical schools today states that “I will apply, for the benefit of the sick, all measures which are required.” The social contract for physicians to improve the health of those who are suffering necessitates broader, societal interventions [28–30]. Clinicians must tackle this challenge by incorporating social concepts into their daily practice and becoming a powerful voice in the public debate. During the Katrina “unnatural disaster,” David Brooks made a simple yet profound observation: “Floods wash away the surface of society, the settled way things have been done. They expose the underlying power structures, the injustices, the patterns of corruption and the unacknowledged inequalities”[31].

## Conclusion

Only systematic change can reverse the disparities that we observe today, to transform the heavy mantle of inequality into action to change the fundamental causation of disease. We need to act on an existing social science knowledge base and add to that foundation through systematic research. It is our professional obligation to address inequities caused by larger social forces, both local and worldwide, which create and perpetuate inequality and poor health.
