# Correction: Health Is Still Social: Contemporary Examples in the Age of the Genome

**DOI:** 10.1371/journal.pmed.0050010

**Published:** 2008-01-29

**Authors:** Timothy H Holtz, Seth M Holmes, Scott Stonington, Leon Eisenberg

Correction for:

Holtz TH, Holmes S, Stonington S, Eisenberg L (2006) Health is still social: Contemporary examples in the age of the genome. PLoS Med 3(10): e419. doi:10.1371/journal.pmed.0030419

One of the authors, Seth M. Holmes, was referred to as Seth Holmes in the author line and in the citation.

The correct citation is: Holtz TH, Holmes SM, Stonington S, Eisenberg L (2006) Health is still social: Contemporary examples in the age of the genome. PLoS Med 3(10): e419. doi:10.1371/journal.pmed.0030419

